# Correlation of Promontory Vibration and Sound Emission Recorded from the Skin Surface in Bone Conduction Stimulation

**DOI:** 10.1007/s10162-025-01028-6

**Published:** 2026-01-20

**Authors:** Mohammad Ghoncheh, Patrick Maas, Nils Prenzler, Rolf B. Salcher, Thomas Lenarz, Hannes Maier

**Affiliations:** 1 Department of Otolaryngology and Cluster of Excellence “Hearing4all”, Hannover Medical School, Carl-Neuberg-Str. 1a, 30625 Hanover, Germany; 2 Formerly Oticon Medical, Smoerum, Denmark

**Keywords:** Bone conduction stimulation, Surface microphone, Bone conduction implant, Laser Doppler vibrometry

## Abstract

**Purpose:**

Sound pressure recordings at different positions at the head such as ear canal, nasal cavity, or on the skin are effective tools to verify, fit, or follow up the output of bone conduction devices (BCDs) intra- and post-operatively. Here, we investigated the possibility of using a surface microphone (SM) as a non-invasive alternative to laser Doppler vibrometry (LDV) measuring cochlear promontory (CP) vibrations in human heads.

**Methods:**

A percutaneous BCD (Ponto system) was implanted at the standard position in five human (four males/one female) cadaver heads (ten ears). CP vibration was measured using LDV in response to the BCD stimulation. Simultaneously, the sound pressure level (SPL) emitted by the skin was measured by the SM attached to the forehead of the specimens. A linear regression model estimated the vibration amplitudes based on the measured SPL.

**Results:**

A frequency-independent linear regression between recorded SM SPL and CP velocity showed a significant correlation (slope = 1.012; *r*^2^ = 0.535, *p* < 0.001). An enforced fixed slope (constant = 166.7 dB) of one resulted in a mean absolute error of MAE = 7.7 ± 2.7 dB across frequencies. Although the initial linear model with a fixed slope of one showed frequency-dependent deviations, applying a frequency-specific correction significantly improved the prediction accuracy (*r*^2^ = 0.557, MAE = 6.5 ± 1.5 dB).

**Conclusion:**

Microphone-based recording of acoustic surface emissions offers a non-invasive alternative to LDV to assess BCD output.

**Supplementary Information:**

The online version contains supplementary material available at 10.1007/s10162-025-01028-6.

## Introduction

Bone conduction devices (BCDs) offer an alternative treatment option for individuals with conductive hearing loss (CHL) or mixed hearing loss (MHL), particularly when conventional hearing aids or middle ear surgeries prove ineffective [1, 2]. Acoustic emissions recorded from different positions at the head are a commonly investigated method to determine the output of bone conduction devices (BCD) in patients clinically. Recordings in response to BCD stimulation from the external auditory canal (EAC), either on the ipsilateral or contralateral side as well as nasal sound pressure, are used to measure non-invasively the output of vibratory bone stimulators in patients [3–5]. However, all these sound recordings in response to BCD stimulation are limited to research purposes and not established procedures in clinical routine. Besides functional testing and the determination of maximum output level, surface microphone recordings are a promising tool for the verification of BCD output levels in clinical routine. This was key for the development of the surface microphone used in our study [6] and had been successfully demonstrated for determination of output audibility and the verification of the implemented fitting rules [7, 8].

On the other side, various methods to measure the output level of BCD have been employed in preclinical experiments. The most commonly used is the one-dimensional (1D) measurement of cochlear promontory (CP) vibration by laser Doppler velocimetry (LDV) as an indicator for output level [9]. However, other elaborate methods that are more difficult to carry out, such as 3D accelerometry at positions close to the cochlea or 3D-LDV, may provide a more detailed picture of cochlear vibration as these methods also take in-plane vibrations into account [10, 11]. Nevertheless, all methods based on CP or skull vibration magnitude are indirect measures of input to the cochlea. Here, differential intra-cochlear sound pressure difference measurements across the basilar membrane are a more direct measure of perceived loudness [12, 13], but are less established, highly invasive, and potentially subject to vibration artefacts [14, 15].

In our study, we intended to explore the potential (linear) relationship between the preclinical standard 1D-LDV measurement on the CP and the clinically applicable surface microphone recordings.

## Method and Material

The use of whole human cadaver heads for these experiments was approved by the ethics committee of the Hannover Medical School (8151_BO_K_2018). This study intends to investigate the possible relationship between the sound pressure recorded from the skin on the forehead and the one-dimensional promontory vibration velocity when the skull is excited by a bone vibrator. In case of a linear relationship, the sound pressure recorded would be:

1 $$P(f) = a(f) v(f)$$

where *P* is the sound pressure, *f* is the frequency, *a*(*f*) is a frequency-dependent factor, and *v*(*f*) is the velocity measured on the promontory at each frequency on the promontory. As the promontory vibration and sound pressure are evoked by the vibratory excitation, a possible constant in Eq. 1 must be zero at all frequencies. In logarithmic domain and in terms of decibel related to m/s (dB re. m/s) for the cochlear promontory velocity $CP_{Velocity}$ and decibel sound pressure level measured with the surface microphone $SM_{SPL}$ [dB SPL] follows:

2 $$SM_{SPL} (f)[dB\;SPL] = A_{0} (f) + CP_{Velocity} (f)[dB\;re\;m/s]$$

where $A_{0} (f) = a(f) + const$ is a frequency-dependent constant. Hence, in the linear case, the measured sound pressure level is related to the measured promontory velocity in dB with a slope of one and a frequency-dependent constant.

### Cadaver Heads

Five fresh frozen human cadaver full heads (four males/one female) were deployed for measurements of cochlear promontory (CP) vibrations and forehead sound radiation in response to vibration stimulation with the percutaneous bone anchored hearing system (BAHS) Ponto 3 (Oticon Medical, Askim, Sweden). Specimens were the same described in a previous publication on output performance of a novel transcutaneous bone conduction implant [16]. The cohort comprised one female and four male head specimens. There was no information on medical or injury history. All heads were scanned with flat-panel cone-beam computed tomography (CT) prior to experiments. Two experienced surgeons (RS, NP) examined the CTs to exclude fractured skulls before experiments. Defrosting of heads took place in a cooling room at + 4 °C at least 24 h before the experiment. The cochlear promontory of both ears was made accessible via a > 2 × 2 mm opening in the tympanic membrane above the promontory. The CP vibration (ipsi- and contralateral) responses and sound pressure measurements on the forehead were simultaneously performed, and experiments were conducted sequentially on both ears during two consecutive days in the five heads, resulting in datasets for ten ears. In between measurements on day 1 and day 2, heads were stored at + 4 °C.

For the determination of linearity of the relationship between CP vibration and SM recording, the attenuation of ambient airborne sound by the surface microphone, an additional human cadaver full head was stimulated with a percutaneous device (Ponto SP, Oticon Medical, Sweden) and a loudspeaker in the sound field (Mackie, HR824mk2, USA) in an anechoic chamber.

### Implantation

A Ponto system (Wide Ponto implant, Oticon Medical, Askim, Sweden) was implanted at the intended percutaneous BAHS implant position approximately 55 mm posterior-superior to the ear channel entrance. An incision was made, and skin was retracted for exposing the skull bone. A 4-mm-long and 4.5-mm-diameter wide implant was placed into the skull bone using the standard guide drill and a 4-mm countersink drill (Oticon Medical, Askim, Sweden). A 9-mm-long abutment was attached to the implant of the Ponto BAHS system (Oticon Medical, Askim, Sweden).

### Stimulation

A modified Ponto 3 sound processor (Oticon Medical, Askim, Sweden) was used for stimulation. Modifications enabled direct electrical stimulation via one microphone port, turning off the other microphone and setting the gain to linear. Before and after each experiment, the sound processor’s performance was verified with a skull-simulator (SKS-10, Interacoustics, Middelfart, Denmark) to ensure its functionality according to specifications.

Stimulation signal synthesis was the same as described in a previous publication [16]. In short, the signal injected to the sound processor was a stepped sine consisting of 77 logarithmically spaced sinusoids from 87.5 Hz to 10 kHz. The signal was generated by a multi-channel data acquisition system (NI-4431 BNC, National Instruments, Austin, TX, USA) with a 24-bit analogue-to-digital converter. The acquisition system was controlled with custom-made control software using LabVIEW (National Instruments, Austin, TX, USA). For providing the required current, a power amplifier (SA1, Tucker Davis Technologies, USA) was connected between the signal generator and the modified sound processor.

### Laser Doppler Measurements

Velocity responses were measured on both ipsi- and contralateral cochlear promontories (CPs) for each stimulation side, but results and analyses shown in this publication originate from the ipsilateral cochlear promontory only. Measurement sites were visually accessed via the small incision in the tympanic membrane, and LDV measurements were obtained from a small reflector placed on the CP to improve signal-to-noise ratio. The laser beam was almost perpendicular to the CP surface.

For the velocity response measurements, a laser Doppler vibrometer (LDV) (OFV 534, Polytech, Germany) mounted on a surgical microscope (OPMI 1, Zeiss, Germany) was used. The LDV system’s sensitivity was set to 5 mm/s/V, and data were sampled at 51.2 kS/s and averaged between 30 and 500 times for ensuring a signal-to-noise ratio (SNR) of a minimum of 12 dB. For the noise floor estimation, the average of six adjacent bins to the stimulation frequency in the computed Fast Fourier transform (FFT) was employed. Data with a SNR below 12 dB were excluded from the data analysis.

### Surface Microphone Measurement

**Fig. 1:**
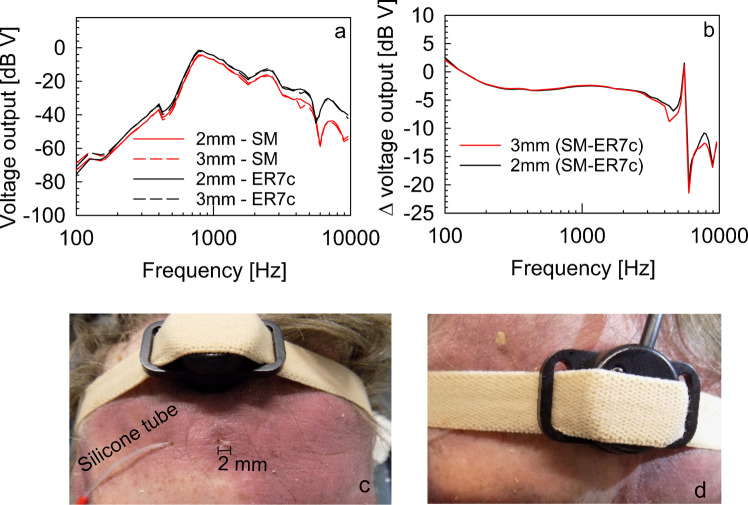
**a** Absolute output voltage of the SM and reference probe microphone ER7c in response to bone-conducted stimulation by the Ponto 3. **b** Difference in output of the SM to the reference sound pressure level as measured approximately 2 mm and 3 mm above the skin surface in the SM cavity. **c** The configuration of the ER7c reference microphone tube guided subcutaneously to the cavity below the SM. **d** The SM held by a headband on the middle of the forehead during the calibration measurement

The employed surface microphone was the next-generation prototype by Interacoustics A/S (Middelfart, Denmark) based on an earlier design by Hodgetts et al. [6]. In our setup, the surface microphone (SM) was attached to the middle of the forehead using an elastic band (Fig. 1) and connected via a preamplifier to the data acquisition input. The recording was performed simultaneously using the same data acquisition system and SNR criterion of ≥ 12 dB as described before for the LDV vibration measurements. The stimulation was done sequentially on both sides, and due to the symmetry with respect to measurement points, data from left and right ears was pooled for the analysis.

The design of the surface microphone follows the principle of a stethoscope. In a conical cavity, structural vibrations of skull and skin tissue at the SM base emit sound into the void. At the apex, a microphone is placed sensing the acoustic sound field [6].

Calibration of the SM output was performed with a calibrated probe microphone (ER7c, Etymotic, IL, USA) placed in the cavity of the SM on the forehead of one cadaver head specimen. The silicone probe tube attached to the reference probe microphone was guided subcutaneously into the SM cavity, with the opening placed approximately 2 mm and 3 mm above the skin (Fig. 1c). Both microphones measured the sound pressure in the surface microphone cavity during BAHS stimulation simultaneously. Figure 1a shows the magnitude response of the surface microphone output and the reference microphone 2 and 3 mm above the skin; the difference between 2 and 3 mm was negligible; therefore, we used 2 mm as reference. The magnitudes’ difference between reference and surface microphone recordings was used to calibrate the output magnitude of the surface microphone (see Fig. 1b, Supplementary Table 1). All SM data in the following analysis are shown calibrated in dB SPL and shown as SM_SPL_.

### Linearity and Attenuation of Ambient Sound

The modified Ponto 3 sound processor (Oticon Medical, Askim, Sweden) was used to investigate the linearity between the measured sound pressure by the SM and the vibration of the cochlear promontory measured by the single-beam LDV in bone conduction stimulation mode. The input voltage for the actuator to test the linearity was between 0.1 mV and 2.43 V.

The performance of the SM in terms of attenuation of the ambient acoustic airborne sound was tested using a loudspeaker (Mackie, HR824mk2, USA) in an anechoic chamber. Therefore, in a single human head specimen, the sound pressure close to the housing of the surface microphone was measured using a probe microphone (ER7C, Etymotic, IL, USA) and compared to the synchronously measured SPLs by the SM at frequencies between 100 Hz and 10 kHz and sound field levels between 101 and 119 dB SPL outside the SM at a distance < 1 cm.

To determine the level of airborne sound emitted by the bone conduction device, the Ponto 3 was driven electrically with an input voltage of 1.0 V and the sound pressure level measured approximately 1 cm next to the SM.

### Statistics

Data visualization and analyses were performed with Matlab (Matlab R2021a, MathWorks) and SigmaPlot 15 (Inpixon, Düsseldorf, Germany). A Shapiro–Wilk test was used to check the normality of the dataset. In case of normally distributed data, a paired Student’s *t*–test was used; in case of non-normal distribution, a Mann–Whitney rank sum test was performed. Statistical tests were performed to investigate the differences (*p* < 0.05) between conditions at each frequency.

## Results

### Linearity SM vs. CP Vibration Measurements

**Fig. 2:**
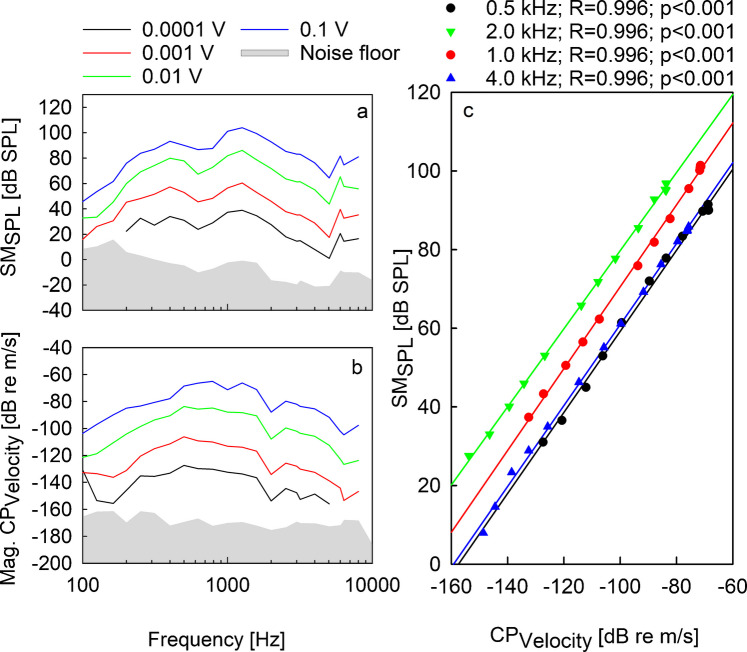
**a** SM sound pressure level (SM_SPL_) and **b** CP vibration (CP_Velocity_) in response to BAHS stimulation with input voltages between 0.1 mV and 0.1 V in a single specimen. The color code for the input voltages is given above panels **a** and **b** and the respective noise floors are depicted as gray shaded areas. **c** Recorded CP velocities and sound pressure levels measured by the SM on the forehead for the full measured input voltage range of 0.1 mV to 2.43 V are plotted for four exemplary frequencies (solid symbols). Linear regressions are shown as solid lines

The linear dependency between the sound pressure measured on the forehead and the promontory vibration amplitude was verified in a separate fresh human head specimen. Below 0.1 V_p_ input to the BC actuator, both CP velocity and SPL responses grew mainly linear and saturated equally at higher input voltages. Figure 2a and b show the recorded sound pressure and CP velocity amplitudes when the Ponto actuator was driven with input voltages between 0.1 mV_p_ and 0.1 V_p_. In Fig. 2c, the obtained CP velocities were measured from 0.1 mV_p_ to 2.4 V_p_ and are plotted against the sound pressures measured with the SM for four exemplary frequencies. Besides the examples depicted in Fig. 2c, sound pressures and CP velocities at all investigated frequencies were correlated statistically significant with *r*^2^-values of > 0.99 (all *p* < 0.001) although the Ponto 3 system went into saturation at high input voltage.

Attenuation of ambient sound by the SM was tested using the loudspeaker and the calibrated microphone on a human head specimen outside the housing of the SM in a sound-proof chamber. The frequency-dependent attenuation is shown in Supplementary Fig. 6a. To estimate the artefact in SM recordings due to a possible airborne pathway, the sound emission of the BAHS was measured with an input voltage of 1.0 V_P_ to the Ponto 3 at a position near the housing of the SM, and the attenuation levels provided by the housing of the SM for acoustic sound field and BC stimulation were determined (see Supplementary Fig. 6b, Table 4).

### SM vs. CP Vibration Measurements

**Fig. 3:**
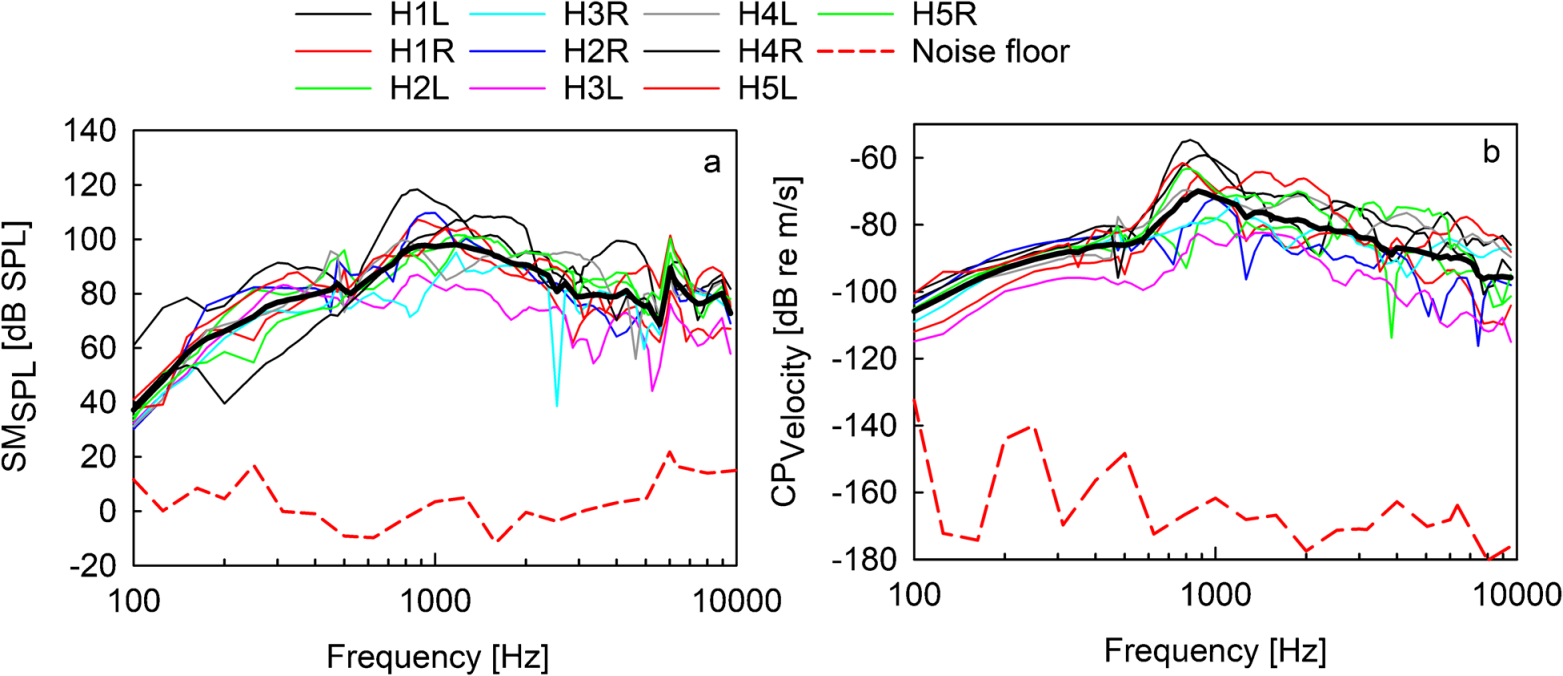
Surface microphone sound pressure level (SM_SPL_) and CP_Velocity_ in response to BAHS stimulation. **a** Sound pressure levels measured by the surface microphone on the forehead with Ponto stimulation. **b** Ipsilateral CP response velocity magnitudes with Ponto stimulation. Solid thin lines represent individual data of each ear, and thick red solid lines illustrate mean values (MV). Dashed red lines depict a representative noise floor example

The sound pressure level measured by the surface microphone on the forehead (SM_SPL_) and the velocity of the ipsilateral CP vibrations (CP_Velocity_) in response to bone-conducted stimulation at an input level equivalent to 90dB SPL (OVFL90) are shown in Fig. 3. Figure 3a shows sound pressure levels measured by the surface microphone in five cadaver heads stimulating sequentially both sides. The recorded average sound pressure level range was between 42 and 93 dB SPL with a maximum at approximately 1 kHz. Figure 3b shows the simultaneously measured CP velocity magnitudes of the same stimuli with average magnitudes ranging from −109 to −70 dB re. m/s. Similar to SM data, a maximum at approximately 1 kHz was observed. SM_SPL_ and CP_Velocity_ mean values (MV) show similar frequency characteristics, suggesting a linear relation between both measurement methods. In the following first step, CP_Velocity_ was assumed as the gold standard representing the true output of the BC stimulus at the cochlear level [17].

**Fig. 4:**
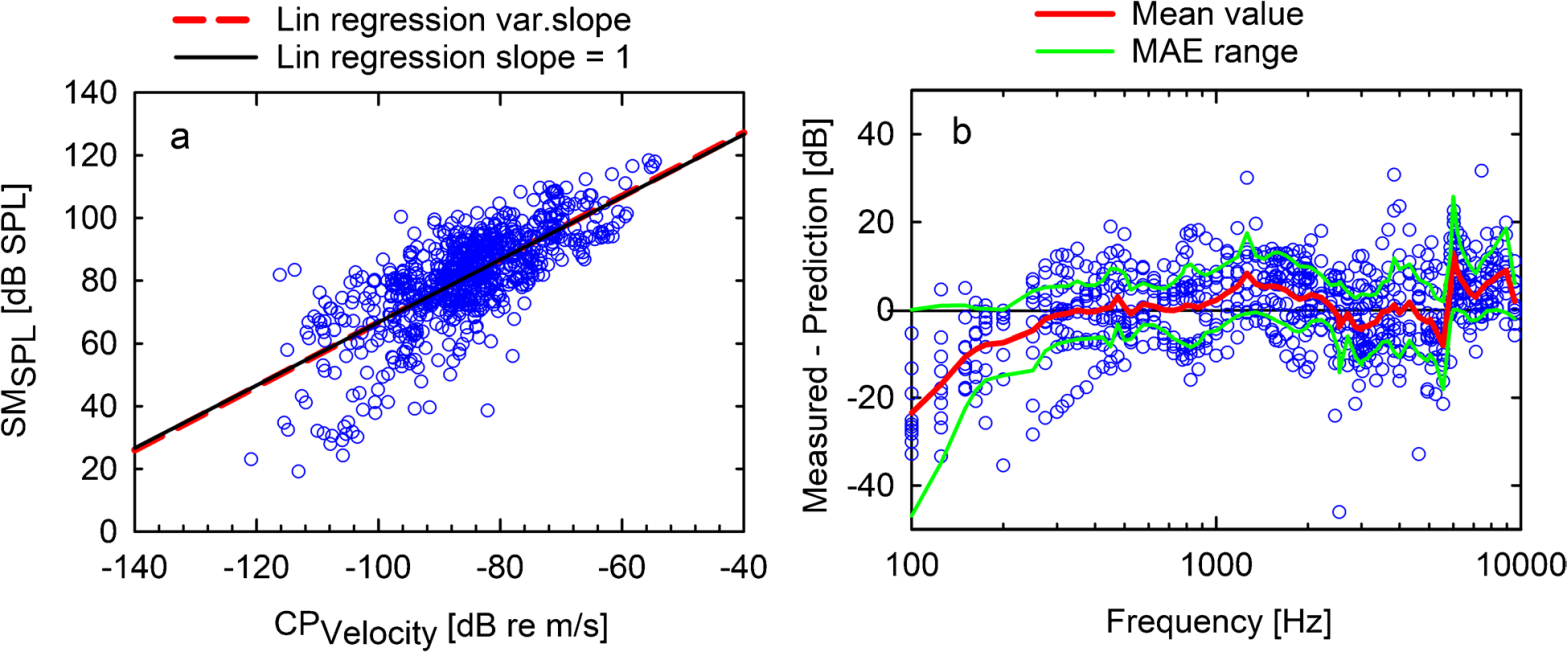
**a** The relation between CP_Velocity_ magnitudes and surface microphone sound pressure levels SM_SPL_ measured on the forehead. The red dashed line shows the linear regression (SM_SPL_ [dB SPL] = 1.012 CP_Velocity_ [dB re m/s] + 167.7 dB). The black solid line depicts the linear approximation with a fixed slope of one (SM_SPL_ [dB SPL] = CPVelocity [dB re m/s] + 166.7 dB). **b** The difference between the measured SM_SPL_ and the linear prediction using a frequency-independent constant Ā_0_=167.7 dB SPL. The red line shows the average deviation (*N* = 10, see emphasis Eq. 4), and green lines depict the MAE range for each frequency. Individual data points are represented by blue circles

To test the hypothesis of a linear dependence, a linear regression was applied to investigate the relation between forehead SM_SPL_ and CP_Velocity_ magnitudes. In this first approach, correlating CP_velocity_ magnitudes against sound pressure levels measured by the surface microphone, the linear relationship was assumed independent of frequency. When pooling all data points across experiments and frequencies, the resulting linear regression slope was close to one (slope = 1.012; *r*^2^ = 0.535, *p* < 0.001, see Fig. 4a). The assumption of a linear relationship between individual forehead sound pressure $P(f)$ and CP velocity $v(f)$ in Eq. 1 was earlier shown in the single head experiment (Fig. 2) that demonstrates a highly significant linearity between SM_SPL_ and CP_Velocity_. However, the linear correlation of the pooled data depends not only on the transfer function between $CP_{Velocity} (f)$ and $SM_{SPL} (f)$ (Eq. 2), but also on two separate factors: the frequency dependence of the constant $A_{0} (f)$ and the intersubject variability between experimental results at each frequency.

The influence of the frequency dependence was addressed in the following approach. Under the assumption of a linear relationship, a fixed slope of one at each frequency *f*_*j*_ was enforced. In this case, any optimized linear approximation necessarily passes through the center of gravity (${CP}_{Velocity} (f_{j} ),\;{SM}_{SPL} (f_{j} )$) leading to:

3 $${SM}_{SPL} \left( {f_{j} } \right) = A_{0} (f_{i} ) + {CP}_{Velocity} \left( {f_{j} } \right)$$

with $${CP}_{Velocity} \left( {f_{j} } \right) = \frac{1}{N}\sum\limits_{i = 1}^{N} {CP_{Velocity}^{i} \left( {f_{j} } \right)}$$ and $${SM}_{SPL} \left( {f_{j} } \right) = \frac{1}{N}\sum\limits_{i = 1}^{N} {SM_{SPL}^{i} \left( {f_{j} } \right)}$$.

where *N* depicts the number of experiments and $CP_{Velocity}^{i} (f_{j} )$ and $SM_{SPL}^{i} (f_{j} )$ are the respective experimental results at frequencies $f_{j}$ with index *j*. In a first step, the constant in Eq. 3 was assumed independent of frequency; $A_{0} (f_{j} ) = \overline{{A_{0} }} = const$ leading to:

4 $$\overline{SM}_{SPL} = \overline{{A_{0} }} + \overline{CP}_{Velocity}$$

with $$\overline{SM}_{SPL} = \frac{1}{M}\sum\limits_{j = 1}^{M} {{SM}_{SPL} (f_{j} )} ;\;\;\;\;\;\;\overline{CP}_{Velocity} = \frac{1}{M}\sum\limits_{j = 1}^{M} {{CP}_{Velocity} (f_{j} )}$$.

where $\overline{SM}_{SPL}$ and $\overline{CP} {}_{Velocity}$ are the gross average magnitudes across *M* measured frequencies. The linear regression of the data pooled across frequencies leads to a slope not significantly different from one (slope *a* = 1.012, confidence interval = [0.95, 1.08], see Fig. 4a). As the linear regression distinguished between an independent and a dependent variable, the enforcement of a slope of one drops the prioritization of $CP_{Velocity} (f)$ as “gold standard” and balances the relationship. A slope of one results in a frequency-independent constant of $\overline{{A_{0} }} = 167.7\;dB$ and is illustrated in Fig. 4a. If correlation analysis is additionally done within single octave bands, the confidence interval of the slope of the correlation contained a slope of one in none of the seven analyzed bands, due to reduced dynamic range of the data (see Fig. 7 and Table 5 in the supplements).

Plotting the deviation of this approximation for each frequency shows that the assumption of a frequency-independent constant leads to a substantial error (Fig. 4b, Supplementary Table 2). Specifically, for frequencies below 275 Hz, the frequency-independent linear model underestimates SM_SPL_ magnitudes, while for mid-frequencies, it overestimates them. The linear, frequency-independent approach with a fixed slope of one results in a root mean square error (RMSE) of 9.4 ± 3.1 dB and a mean absolute error (MAE) of 7.7 ± 2.7 dB across frequencies. In contrast, the linear regression with variable slope leads to a similar RMSE = 9.4 ± 3.0 dB and MAE = 7.7 ± 2.7 dB, supporting the assumption of a linear relationship.

However, the linear relationship with a slope of one in the logarithmic domain and a frequency-independent constant still shows a pronounced frequency dependence of the deviation between the prediction and measured sound pressure levels (Fig. 4b). For this reason, in a refined approach, the frequency dependence (Eq. 3) of the constant was determined using the average emphasis ${E}(f_{j} )$:

5 $${SM}_{SPL} \left( {f_{j} } \right) = \overline{{A_{0} }} +{E}(f_{i} ) + {CP}_{Velocity} \left( {f_{j} } \right)$$

**Fig. 5:**
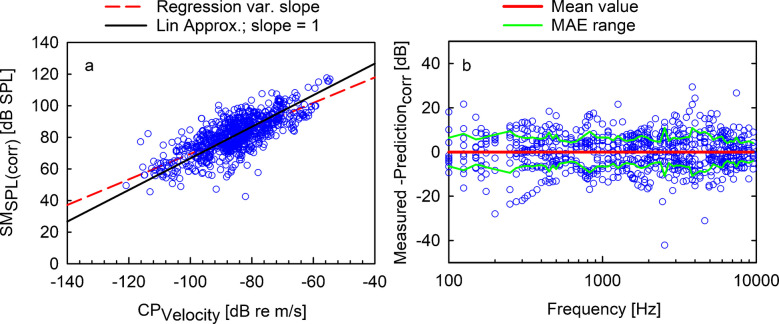
**a** The relation between the CP_Velocity_ magnitudes and SM_SPL_ measurement results on the forehead. For better visualization, emphasis-corrected surface microphone recordings ($SM_{SPL\left(corr\right)}^i\left(f_j\right)=SM_{SPL}^i\left(f_j\right)-E\left(f_j\right)$) are shown (blue circles). The red dashed line shows the linear regression (SM_SPL(corr)_ [dB SPL] = CPVelocity [dB re m/s].0.808 + 150.2 dB; *r*^2^ = 0.557), and the black solid line depicts the linear approximation with a slope of one of the pooled emphasis corrected data. **b** The difference of the measured and predicted surface microphone values. The red line shows the average, and the green lines depicts the MAE range. Individual data points are represented by blue circles

Average emphasis ${E}(f_{j} )$ across experiments was calculated from ${CP}_{Velocity} (f_{j} ),\;{SM}_{SPL} (f_{j} )$, and $\overline{{A_{0} }} = 166.7\;dB$ for each frequency *j* and are listed in Supplementary Table 3. Figure 5a shows CP_Velocity_ magnitudes against the emphasis-corrected SM_SPL(corr)_ results. A linear regression results in (SM_SPL(corr)_ [dB SPL] = 0.808 CP_Velocity_ [dB re m/s) + 150.2 dB, *r*^2^ = 0.557, *p* < 0.001). By the linear approximation $SM_{SPL}^{i} \left( {f_{j} } \right) = \overline{{A_{0} }} +{E}(f_{i} ) + CP_{Velocity}^{i} \left( {f_{j} } \right)$ with a slope of one, using $\overline{{A_{0} }} = 166.7\;dB$ and the frequency-specific average emphasis ${E}(f_{j} )$ from Supplementary Table 3, the average deviation of the prediction at each frequency is set to zero (see Fig. 5b). In comparison with the previous method, namely the frequency-independent linear prediction, both the root mean square error (RMSE) = 7.9 ± 2.0 dB and the mean absolute error (MAE) = 6.5 ± 1.5 dB across frequencies are substantially reduced. Additionally, the distribution of the difference between the individual SM estimates from the promontory vibration shows a homogeneous normal distribution (Shapiro–Wilk) around the reference, exceeding rarely a MAE of 10 dB (2537.5 and 3837.5 Hz) in the frequency range between 87.5 Hz and 10 kHz.

## Discussion

Surface microphone recordings have been demonstrated to be a useful tool for the verification of BCDs’ output in patients [6]. The purpose of our study was to investigate the possible relationship of this non-invasive, clinically usable method to a commonly used LDV as an established experimental method that is mainly limited to preclinical testing due to its invasiveness. We have compared SM recordings on the forehead to 1D-LDV measurements on the cochlear promontory in ten ears of five human cadaver heads. This study did not perform a gender-based analysis, as it was beyond the scope of the present investigation. Our focus here was to show the possible equivalence of both methods, their relative accuracy, variability, and frequency dependence of the transfer function and to provide reference data for quantitative conversion.

In a first step, the frequency characteristics of the surface microphone were verified under realistic conditions on human skin against a calibrated probe microphone close to the sound emitting surface in a single cadaver head. Below 4 kHz, the response of the SM was flat with deviations < 5 dB from the reference (Fig. 1). A resonance between 5 and 6 kHz, however, led to pronounced deviations compared to the reference microphone recorded close to the surface. The necessary correction (Fig. 1, Supplemental Table 1) of all sound pressure levels recorded by the SM was done at all frequencies.

A linear relationship of force input to acceleration output was already demonstrated in a male subject [18]. Our verification of linearity between CP vibration and SM recording in a single specimen demonstrated that both measures are linearly correlated with a slope of one (*R*^2^ > 0.99; *p* < 0.001) at all frequencies (Fig. 2). In our experiments, the dynamic range covered approximately 80 dB, and the linear relationship was insensitive to BC transducer saturation. Although the slope indicated a highly linear relationship at each frequency, it was apparent from the regressions across frequencies that the transfer function has a mild frequency dependency (Fig. 4b, Eq. 3). Moreover, the linear correlation in the pooled data of the ten ears across frequencies was even less pronounced (Fig. 4a), probably due to inter-individual differences and the frequency dependence. An analysis in octave bands reduces the respective CP input ranges and consequently decreases the correlation (see Supplementary Information Fig. 7; Table 5).

A subsequent refined frequency-specific linear regression yields an estimate of the frequency dependence of the constant $A_{0} (f)$ in Eq. 2 (Fig. 4b), adding roughly ± 10 dB emphasis between 0.1 and 10 kHz (see Supplementary Table 2). Integration of the frequency dependence of the constant according to Eq. 5 leads to normally distributed SM_SPL_ estimates with a homogeneous distribution around the reference with a MAE of usually < 10 dB. This linear prediction, including the frequency dependence with a mean absolute error of MAE = 6.5 ± 1.5 dB across frequencies, provides a good estimate of cochlear promontory vibration from surface microphone measurements on the forehead and is sufficiently accurate for many practical purposes. The variability of SPLs and CP vibration recording is comparable in size as can be expected from inter-individual variability in similar well-controlled preclinical experiments [9, 16, 19]. Furthermore, the test–retest reliability of SM and CP was excellent (data not shown) and variability between recordings could be excluded. Although, the number of experiments showing the linear relationship between SM recording and CP velocity on an individual basis was limited in our present work, it is likely that the deviation of the regression from a slope of one in the pooled data (Fig. 5) originates in inter-individual differences. This supports earlier findings demonstrating linearity between audiometric BC thresholds and BC vibrator input [20] as well as in transcranial transmission [18]. Nevertheless, extending the evidence for linearity in more specimens and in the dynamic range investigated here would be desirable in future studies.

It is further worth to note that in the present work, 1D-LDV CP velocity was first assumed as “gold standard” to determine the MAE/RSME in the prediction from surface microphone measurements and given up in the final analysis. As a consequence of an enforced condition of a slope = 1, this relationship is symmetric; i.e., when estimating CP vibrations from surface microphone recordings, identical MAEs and RMSEs apply.

For BCDs in general, but in particular after the introduction of transcutaneous BCDs, that are inaccessible for testing after implantation, objective state-of-the-art measurement methods for testing functionality are indispensable. Methods to test audibility, fitting targets in situ, and technical integrity intraoperatively as well as clinical verification of results are commonly not implemented in clinical routine. To determine the output of BCDs, ear canal sound pressure (ECSP) either on the ipsilateral or contralateral side has been investigated by different research groups [3, 4]. However, anatomic and surgical disadvantages are the proximity of the measurement site to the surgical site and some patients suffering from ear canal atresia do not have ear canals [5]. As an alternative, nasal sound pressure (NSP) measurements were introduced for functionality testing and verification of fitting of transcutaneous bone conduction systems as well as tracking coupling efficiency over time [5]. As patients’ breathing can affect measurement and some patients may not be asked to hold breath for the duration of the measurement, e.g., in anesthesia, nasal breathing can impede NSP measurements [21]. Laser Doppler vibrometry on the other hand is typically used in cadaver specimens, although Eeg-Olofsson et al. have shown its feasibility in patients with unilateral middle ear common cavity, during stimulation by bone conduction [17]. This approach allows a direct comparison of preclinical and clinical results but will be limited to only a fraction of patients and is unlikely to be part of clinical routine. In contrast, surface microphone recordings on the forehead are mainly independent from most constraints and can be easily implemented under sterile covers during surgeries as well as in clinical audiology settings. They also allow better shielding from ambient sound, especially when sound field stimulation is required for verification of fitting targets or determination of audibility [6, 8].

Moreover, SM recordings potentially entail an additional possible advantage for the applications of upcoming and clinically established transcutaneous BCDs. As transcutaneous BCDs have fewer positioning restrictions; i.e., the separation of processor/microphone position from the stimulation site becomes a feasible option to increase stimulation efficiency for bone conduction sites positioned closer to the cochlea [9, 16, 22, 23].

CP velocity using 1D-LDV as investigated here is the most commonly used measurement method, as it is relatively simple, combining several advantages, e.g., the defined measurement direction along the ear canal, approximately perpendicular to the skull surface, with the avoidance of a posterior tympanotomy surgery to reach the middle ear cavity.

1D-LDV measures the vibration only in one direction, i.e., in line with the EAC at the cochlear promontory. As the cochlea is sensitive to vibrations in all directions, this can be a limiting factor when estimating the real hearing sensitivity from a single vibration direction. The stimulation direction in a bone conduction hearing device is perpendicular to the skull bone and approximately in line with the measurement direction along the EAC; this direction might be the most important contributor to perceived sound using a BAHA or a BCD [10, 22, 24].

However, it cannot be excluded that 1D-LDV undergoes only quantitative changes with stimulation at alternative positions, but also qualitative changes, i.e., an altered vibration direction, such as off-axis vibrations and stimulation position-dependent changes in vibration excitation [25, 26]. According to Stenfelt et al., vibration measurements of the cochlea in one direction are appropriate for frequencies < 3 kHz, while three-dimensional vibration data give better estimates at higher frequencies [22, 24]. Methods including surface microphone recordings that integrate vibrational responses of longitudinal and transversal components across three dimensions, like 3D accelerometry or 3D-LDV, are potentially more adequate to mirror human hearing perception. Although the latter aspect is highly speculative, further research appears worth the effort to be investigated in the future as surface microphone recordings might provide a more general proxy for human BC hearing perception in experiments and clinical routine.

## Conclusion

Recordings of acoustic surface emissions with a microphone are non-invasive alternative to laser Doppler velocimetry measurements on the cochlear promontory through the ear canal for output quantification and verification of fitting targets of bone conduction device. Here, we demonstrated the equivalence of both methods in a human cadaver head study of ten ears/five heads and the resulting accuracy. By using a frequency dependence compensation of the transfer function, results from one method can be converted into the other with a root mean square error of RMSE = 7.9 ± 2.0 dB and a mean absolute error of MAE = 6.5 ± 1.5 dB.

## Supplementary Information

Below is the link to the electronic supplementary material.

ESM 1 (DOCX 582 KB)

## Data Availability

The authors confirm that the data supporting the outcomes of this study are available within the article and its supplementary materials. Raw data and derived data supporting the findings of this study are available from the corresponding author (HM) on request.
