# Supplementary material for: Correlation of Promontory Vibration and Sound Emission Recorded from the Skin Surface in Bone Conduction Stimulation
Source: J Assoc Res Otolaryngol. 2026 Jan 20;27(2):263–72. doi: 10.1007/s10162-025-01028-6 (PMC13090456; doi:10.1007/s10162-025-01028-6)
Supplement: Supplementary file 1 — (DOCX 582 KB) [file 10162_2025_1028_MOESM1_ESM.docx]

# **Supplemental Material**

Table 1 Comparison of voltage output between the surface microphone and the ER7c probe microphone on the human head specimen.

| Freq. [Hz] | 100 | 125 | 150 | 163 | 175 | 200 | 250 | 275 |  |  |
| --- | --- | --- | --- | --- | --- | --- | --- | --- | --- | --- |
| Diff. [dB] | 2.5 | 0.5 | -0.7 | -1.1 | -1.6 | -2.2 | -2.8 | -3.0 |  |  |
| Freq. [Hz] | 300 | 313 | 325 | 350 | 375 | 400 | 425 | 450 | 475 | 500 |
| Diff. [dB] | -3.1 | -3.1 | -3.1 | -3.0 | -3.1 | -3.2 | -3.3 | -3.3 | -3.3 | -3.3 |
| Freq. [Hz] | 525 | 575 | 625 | 675 | 725 | 775 | 788 | 825 | 875 | 925 |
| Diff. [dB] | -3.3 | -3.2 | -3.1 | -3.0 | -2.9 | -2.8 | -2.8 | -2.7 | -2.6 | -2.6 |
| Freq. [Hz] | 1000 | 1088 | 1175 | 1263 | 1350 | 1438 | 1525 | 1588 | 1613 | 1700 |
| Diff. [dB] | -2.5 | -2.5 | -2.5 | -2.5 | -2.6 | -2.6 | -2.7 | -2.8 | -2.8 | -3.0 |
| Freq. [Hz] | 1788 | 1875 | 1963 | 2000 | 2050 | 2213 | 2375 | 2525 | 2538 | 2700 |
| Diff. [dB] | -3.0 | -3.0 | -3.1 | -3.1 | -3.1 | -3.2 | -3.3 | -3.4 | -3.4 | -3.5 |
| Freq. [Hz] | 2863 | 3000 | 3025 | 3175 | 3188 | 3350 | 3513 | 3675 | 3838 | 4000 |
| Diff. [dB] | -3.7 | -4.1 | -4.2 | -4.5 | -4.5 | -4.6 | -4.8 | -5.1 | -5.4 | -5.6 |
| Freq. [Hz] | 4313 | 4625 | 4938 | 5038 | 5250 | 5563 | 5875 | 6000 | 6188 | 6350 |
| Diff. [dB] | -6.0 | -6.9 | -6.2 | -5.7 | -4.2 | 1.5 | -13.2 | -20.4 | -16.6 | -15.4 |
| Freq. [Hz] | 6500 | 6812 | 7125 | 7438 | 7750 | 8000 | 8350 | 8950 | 9550 |  |
| Diff. [dB] | -14.9 | -13.9 | -12.7 | -11.6 | -10.9 | -11.0 | -12.2 | -16.4 | -12.9 |  |

**Table 2:** Mean experimental results and linear predicted values assuming a slope of one and a frequency independent constant of 166.7 dB. The emphasis $\widehat{E}(f_{j})$ was calculated as the mean difference to the linear prediction at each frequency.

| **Freq.** | **SM** | **CP** | **Emphasis**$\widehat{E}(f_{j})$ | **RMSE** | **MAE** |  | **Freq.** | **SM** | **CP** | **Emphasis** $\widehat{E}(f_{j})$ | **RMSE** | **MAE** |
| --- | --- | --- | --- | --- | --- | --- | --- | --- | --- | --- | --- | --- |
| **[Hz]** | **[dB SPL]** | **[dB m/s]** | **[dB SPL]** | **[dB SPL]** | **[dB SPL]** |  | **[Hz]** | **[dB SPL]** | **[dB m/s]** | **[dB SPL]** | **[dB SPL]** | **[dB SPL]** |
| 88 | 31.0 | -109.4 | -26.4 | 27.7 | 26.4 |  | 1788 | 91.3 | -78.7 | 3.3 | 9.0 | 8.0 |
| 100 | 37.2 | -105.8 | -23.7 | 25.0 | 23.7 |  | 1875 | 90.8 | -78.5 | 2.5 | 8.3 | 7.5 |
| 125 | 48.0 | -101.5 | -17.1 | 20.0 | 18.0 |  | 1963 | 90.7 | -78.9 | 2.9 | 8.2 | 7.1 |
| 150 | 58.1 | -97.8 | -10.8 | 13.0 | 11.8 |  | 2006 | 90.6 | -79.3 | 3.1 | 8.0 | 6.7 |
| 163 | 61.3 | -96.3 | -9.1 | 11.5 | 9.4 |  | 2050 | 90.3 | -80.0 | 3.5 | 7.7 | 6.2 |
| 175 | 63.6 | -95.1 | -8.0 | 11.5 | 8.2 |  | 2213 | 88.3 | -81.2 | 2.9 | 7.2 | 5.1 |
| 200 | 66.2 | -93.0 | -7.5 | 12.9 | 7.5 |  | 2375 | 86.7 | -80.9 | 0.9 | 5.4 | 4.7 |
| 250 | 71.6 | -90.3 | -4.8 | 12.4 | 9.0 |  | 2456 | 83.4 | -81.7 | -1.6 | 9.7 | 7.5 |
| 275 | 75.2 | -89.4 | -2.1 | 9.9 | 7.2 |  | 2538 | 80.8 | -82.0 | -3.9 | 16.0 | 10.2 |
| 300 | 76.8 | -88.6 | -1.3 | 9.4 | 6.5 |  | 2700 | 83.8 | -82.0 | -0.9 | 6.0 | 5.0 |
| 313 | 77.2 | -88.4 | -1.1 | 9.1 | 6.4 |  | 2863 | 80.6 | -82.2 | -3.9 | 9.1 | 6.6 |
| 325 | 77.8 | -88.0 | -0.9 | 8.8 | 6.3 |  | 2944 | 78.9 | -83.4 | -4.4 | 9.4 | 8.0 |
| 350 | 78.4 | -88.2 | -0.1 | 8.9 | 6.6 |  | 3025 | 78.9 | -83.8 | -3.9 | 8.4 | 7.5 |
| 375 | 79.2 | -87.0 | -0.5 | 7.6 | 6.0 |  | 3106 | 79.0 | -84.0 | -3.7 | 7.8 | 7.0 |
| 400 | 79.9 | -86.4 | -0.4 | 7.0 | 6.0 |  | 3188 | 79.3 | -84.0 | -3.4 | 7.4 | 6.7 |
| 425 | 80.7 | -86.2 | 0.2 | 7.5 | 6.5 |  | 3350 | 79.6 | -84.3 | -2.8 | 7.9 | 6.4 |
| 450 | 81.4 | -85.8 | 0.5 | 10.3 | 8.8 |  | 3513 | 79.6 | -86.6 | -0.5 | 8.0 | 6.8 |
| 475 | 83.6 | -86.0 | 2.9 | 7.8 | 6.3 |  | 3675 | 79.1 | -87.4 | -0.2 | 8.1 | 6.9 |
| 500 | 81.3 | -86.1 | 0.7 | 8.8 | 7.6 |  | 3838 | 78.8 | -89.0 | 1.1 | 13.7 | 10.6 |
| 525 | 80.1 | -85.6 | -1.0 | 6.2 | 5.6 |  | 4000 | 79.0 | -86.9 | -0.8 | 11.9 | 9.9 |
| 575 | 83.7 | -84.4 | 1.3 | 5.7 | 4.7 |  | 4313 | 81.1 | -87.3 | 1.7 | 9.4 | 8.6 |
| 625 | 86.8 | -80.8 | 0.9 | 4.8 | 4.3 |  | 4625 | 77.4 | -87.7 | -1.7 | 12.3 | 8.4 |
| 675 | 88.8 | -77.9 | 0.1 | 5.4 | 4.8 |  | 4938 | 75.5 | -88.4 | -2.8 | 10.4 | 8.8 |
| 725 | 91.0 | -75.7 | 0.0 | 6.7 | 6.3 |  | 5094 | 75.7 | -88.7 | -2.3 | 8.8 | 7.9 |
| 775 | 94.7 | -72.5 | 0.5 | 8.7 | 8.0 |  | 5250 | 73.0 | -89.1 | -4.6 | 8.8 | 7.3 |
| 800 | 95.4 | -72.5 | 1.1 | 9.7 | 8.7 |  | 5563 | 68.7 | -89.9 | -8.1 | 11.4 | 9.9 |
| 825 | 96.6 | -71.3 | 1.1 | 10.8 | 9.4 |  | 5875 | 82.2 | -89.3 | 4.8 | 9.5 | 7.6 |
| 875 | 97.5 | -69.9 | 0.7 | 9.4 | 8.1 |  | 6031 | 89.5 | -90.1 | 12.9 | 14.8 | 12.9 |
| 925 | 97.8 | -70.5 | 1.5 | 7.7 | 6.5 |  | 6188 | 85.7 | -89.8 | 8.9 | 10.5 | 8.9 |
| 1000 | 97.2 | -71.8 | 2.3 | 7.8 | 6.9 |  | 6344 | 83.9 | -89.8 | 7.0 | 8.8 | 7.4 |
| 1088 | 97.8 | -72.9 | 4.1 | 8.4 | 6.9 |  | 6500 | 82.8 | -89.9 | 6.0 | 7.8 | 6.5 |
| 1175 | 98.1 | -74.1 | 5.4 | 8.6 | 6.8 |  | 6812 | 79.8 | -90.6 | 3.7 | 6.8 | 6.1 |
| 1263 | 97.3 | -77.8 | 8.4 | 12.5 | 9.3 |  | 7125 | 77.9 | -91.7 | 2.9 | 6.8 | 5.2 |
| 1350 | 96.4 | -76.3 | 6.0 | 9.0 | 6.5 |  | 7438 | 76.3 | -95.0 | 4.6 | 10.7 | 6.1 |
| 1438 | 95.7 | -76.3 | 5.3 | 7.7 | 6.4 |  | 7750 | 76.5 | -96.2 | 6.0 | 8.0 | 6.2 |
| 1525 | 95.0 | -77.2 | 5.5 | 8.4 | 7.4 |  | 8050 | 77.5 | -95.5 | 6.4 | 7.9 | 6.6 |
| 1569 | 94.1 | -78.0 | 5.3 | 8.6 | 7.9 |  | 8350 | 78.6 | -95.7 | 7.5 | 9.3 | 7.5 |
| 1613 | 93.8 | -78.1 | 5.2 | 8.6 | 8.0 |  | 8950 | 80.1 | -95.5 | 8.9 | 10.9 | 9.5 |
| 1700 | 92.4 | -78.8 | 4.5 | 8.9 | 7.9 |  | 9550 | 72.8 | -95.8 | 1.9 | 5.1 | 4.0 |

**Table 3:** Mean RMSE and MAE as a function of frequency assuming a slope of one and a frequency dependent constant of 166.7 dB SPL + $\widehat{E}(f_{j})$ (from Table 2).

| **Freq.** | **RMSE** | **MAE** |  | **Freq.** | **RMSE** | **MAE** |
| --- | --- | --- | --- | --- | --- | --- |
| **[Hz]** | **[dB SPL]** | **[dB SPL]** |  | **[Hz]** | **[dB SPL]** | **[dB SPL]** |
| 88 | 8.6 | 7.0 |  | 1788 | 8.3 | 7.3 |
| 100 | 8.0 | 6.5 |  | 1875 | 7.9 | 7.0 |
| 125 | 10.4 | 8.2 |  | 1963 | 7.7 | 6.5 |
| 150 | 7.1 | 5.7 |  | 2006 | 7.4 | 6.1 |
| 163 | 7.0 | 6.1 |  | 2050 | 6.8 | 5.5 |
| 175 | 8.2 | 6.9 |  | 2213 | 6.6 | 4.9 |
| 200 | 10.4 | 7.8 |  | 2375 | 5.3 | 4.6 |
| 250 | 11.4 | 9.4 |  | 2456 | 9.6 | 7.7 |
| 275 | 9.7 | 7.2 |  | 2538 | 15.5 | 11.0 |
| 300 | 9.3 | 6.6 |  | 2700 | 5.9 | 5.0 |
| 313 | 9.1 | 6.4 |  | 2863 | 8.3 | 6.3 |
| 325 | 8.8 | 6.3 |  | 2944 | 8.2 | 6.5 |
| 350 | 8.9 | 6.6 |  | 3025 | 7.5 | 6.0 |
| 375 | 7.6 | 6.1 |  | 3106 | 6.8 | 6.2 |
| 400 | 7.0 | 6.0 |  | 3188 | 6.6 | 6.0 |
| 425 | 7.5 | 6.5 |  | 3350 | 7.4 | 6.4 |
| 450 | 10.3 | 8.9 |  | 3513 | 8.0 | 6.8 |
| 475 | 7.3 | 6.1 |  | 3675 | 8.1 | 6.8 |
| 500 | 8.8 | 7.7 |  | 3838 | 13.7 | 10.9 |
| 525 | 6.1 | 5.3 |  | 4000 | 11.9 | 9.9 |
| 575 | 5.6 | 4.9 |  | 4313 | 9.3 | 8.6 |
| 625 | 4.7 | 4.3 |  | 4625 | 12.2 | 9.1 |
| 675 | 5.4 | 4.8 |  | 4938 | 10.0 | 8.0 |
| 725 | 6.7 | 6.3 |  | 5094 | 8.5 | 7.0 |
| 775 | 8.7 | 7.9 |  | 5250 | 7.5 | 5.5 |
| 800 | 9.6 | 8.5 |  | 5563 | 8.0 | 6.4 |
| 825 | 10.8 | 9.2 |  | 5875 | 8.2 | 7.0 |
| 875 | 9.3 | 7.9 |  | 6031 | 7.3 | 6.9 |
| 925 | 7.6 | 6.5 |  | 6188 | 5.7 | 5.2 |
| 1000 | 7.5 | 6.5 |  | 6344 | 5.4 | 4.6 |
| 1088 | 7.4 | 6.6 |  | 6500 | 5.0 | 4.0 |
| 1175 | 6.7 | 5.2 |  | 6812 | 5.7 | 5.0 |
| 1263 | 9.2 | 7.3 |  | 7125 | 6.2 | 4.6 |
| 1350 | 6.7 | 5.7 |  | 7438 | 9.7 | 6.6 |
| 1438 | 5.6 | 5.0 |  | 7750 | 5.3 | 4.5 |
| 1525 | 6.3 | 5.4 |  | 8050 | 4.6 | 4.1 |
| 1569 | 6.7 | 5.4 |  | 8350 | 5.4 | 4.4 |
| 1613 | 6.9 | 5.6 |  | 8950 | 6.3 | 4.9 |
| 1700 | 7.7 | 6.7 |  | 9550 | 4.8 | 4.1 |


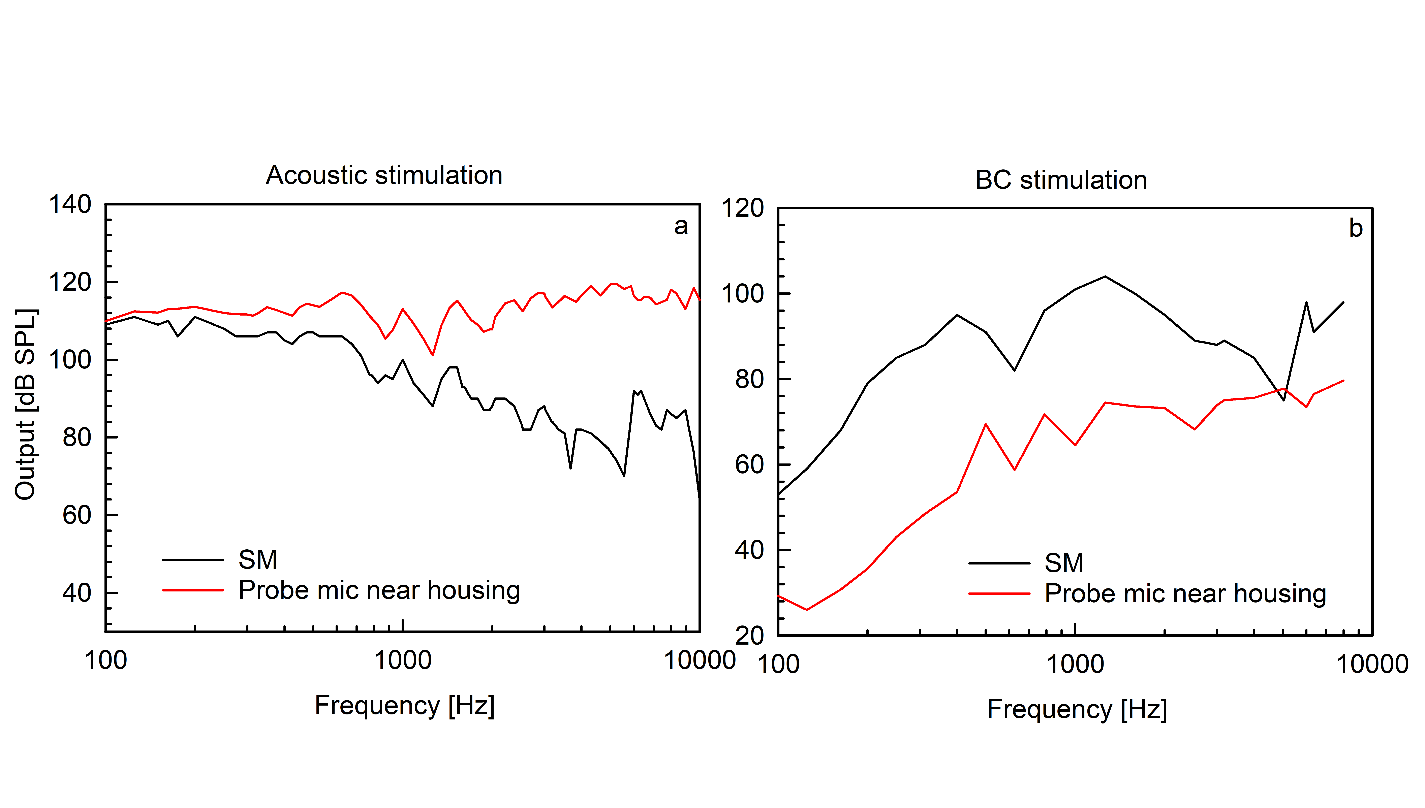


Figure 6: (a) Sound field stimulation in sound proof booth. (b) Ponto stimulation. Both recordings were performed with the surface microphone and the probe microphone recording close to the surface microphone on the forehead.

Table 4: SM housing attenuation

| **Frequency** | **Ponto @1 V** | **Loud speaker @ 1 V** |
| --- | --- | --- |
| **[Hz]** | **SM - ER7C Mic [dB]** | **SM - ER7C Mic [dB]** |
| 100 | -24 | -1.0 |
| 125 | -33 | -1.4 |
| 162.5 | -38 | -2.9 |
| 200 | -44 | -2.6 |
| 250 | -42 | -4.3 |
| 312.5 | -40 | -5.5 |
| 400 | -41 | -6.8 |
| 500 | -22 | -7.0 |
| 625 | -23 | -11.3 |
| 787.5 | -25 | -14.9 |
| 1000 | -37 | -12.9 |
| 1262.5 | -30 | -12.7 |
| 1587.5 | -27 | -20.0 |
| 2000 | -22 | -20.2 |
| 2525 | -21 | -29.1 |
| 3000 | -14 | -29.0 |
| 3175 | -14 | -29.3 |
| 4000 | -9 | -34.6 |
| 5037.5 | 2 | -43.7 |
| 6000 | -25 | -24.9 |
| 6350 | -14 | -23.1 |
| 8000 | -19 | -32.3 |


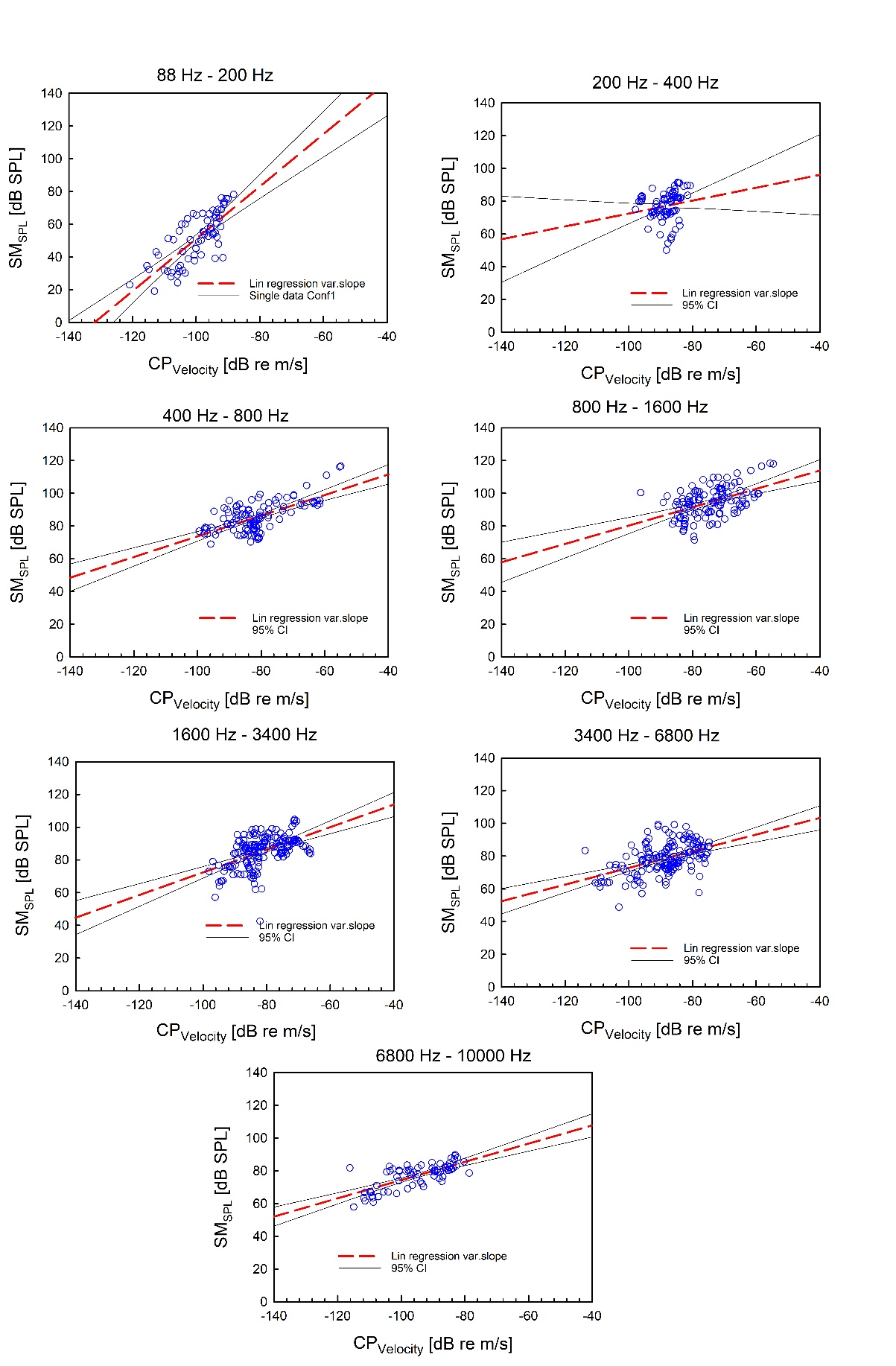


Figure 7: Octave band linear regression analysis. At all octave bands the regression slopes and constants were significantly different from zero (F-test; p < 0.0001) except the slope of second frequency band (p=0.12).

Table 5: The linear regression parameters acquired from the octave band analysis using SM_SPL_ and CP_Velocity_ Pooled data.

| **Freq. [Hz]** | **R^2^** | **f = y_0_+a*x** | **Coefficients** | **Std. Error** | **t** | **P** | **CI Lower Limit** | **CI Upper Limit** |
| --- | --- | --- | --- | --- | --- | --- | --- | --- |
| 88-200 | 0.57 | y_0_ | 210.98 | 17.27 | 12.21 | <0.0001 | 176.47 | 245.48 |
|  |  | a | 1.60 | 0.17 | 9.27 | <0.0001 | 1.25 | 1.94 |
| 200-400 | 0.03 | y_0_ | 111.89 | 22.53 | 4.97 | <0.0001 | 67.04 | 156.75 |
|  |  | a | 0.39 | 0.26 | 1.54 | 0.13 | -0.11 | 0.90 |
| 400­­­-800 | 0.42 | y_0_ | 136.82 | 5.82 | -24.83 | <0.0001 | 125.29 | 148.35 |
|  |  | a | 0.63 | 0.07 | 1.54 | <0.0001 | 0.49 | 0.77 |
| 800-1600 | 0.23 | y_0_ | 136.39 | 7.03 | 23.52 | <0.0001 | 122.47 | 150.30 |
|  |  | a | 0.56 | 0.09 | 8.88 | <0.0001 | 0.37 | 0.75 |
| 1600-3400 | 0.26 | y_0_ | 141.41 | 7.29 | 19.41 | <0.0001 | 127.02 | 155.79 |
|  |  | a | 0.69 | 0.09 | 5.97 | <0.0001 | 0.51 | 0.87 |
| 3400-6800 | 0.22 | y_0_ | 123.71 | 6.74 | 18.35 | <0.0001 | 110.39 | 137.02 |
|  |  | a | 0.51 | 0.08 | 6.73 | <0.0001 | 0.36 | 0.66 |
| 6800-10000 | 0.53 | y_0_ | 130.01 | 6.07 | 21.42 | <0.0001 | 117.90 | 142.12 |
|  |  | a | 0.56 | 0.06 | 8.76 | <0.0001 | 0.43 | 0.68 |

*Std: Standard; CI: confidence interval.
